# Commentary: ATP: The crucial component of secretory vesicles: Accelerated ATP/insulin exocytosis and prediabetes

**DOI:** 10.3389/fphys.2017.00053

**Published:** 2017-02-02

**Authors:** Chitharanjan Duvoor, Vijaya S. Dendi, Asween Marco, Nawal S. Shekhawat, Aditya Chada, Rahul Ravilla, Chaitanya K. Musham, Wasique Mirza, Arun Chaudhury

**Affiliations:** ^1^Department of Endocrinology and Internal Medicine, University of Arkansas for Medical SciencesLittle Rock, AR, USA; ^2^GIM FoundationLittle Rock, AR, USA; ^3^Department of Internal Medicine and Hospital Medicine, Christus Trinity Mother Frances HospitalTyler, TX, USA; ^4^Department of Policy, University of Arkansas for Little RockLittle Rock, AR, USA; ^5^Tutwiler ClinicTutwiler, MS, USA; ^6^Department of Pulmonary and Critical Care Medicine, University of Arkansas for Medical SciencesLittle Rock, AR, USA; ^7^Winthrop P. Rockefeller Cancer Institute, University of Arkansas for Medical SciencesLittle Rock, AR, USA; ^8^St. Vincent Infirmary (Catholic Health Initiative)Little Rock, AR, USA; ^9^The Wright CenterScranton, PA, USA

**Keywords:** exocytosis, SLC17A9, diabetes mellitus, cell secretion, ATP, purinergic

We are aware of concepts of osmotic and oncotic pressure in clinical physiology. Per Starling's law, the freely movable ions do not significantly contribute to the retention of vascular volume, or the maintenance of interstitial fluid pressure (Jacob and Chappell, 2013). On the other hand, albumin, due to its molecular size and inefficiency of free diffusion through the transcellular pores, assumes a major role of maintaining oncotic pressure and retaining fluid within the vascular space. The implications are obvious: either the lack of protein synthesis in liver diseases or loss of protein through the leaky glomerulus in nephrotic conditions leads to deficiency of oncotic pressure, fluid leak and its accumulation in tissue spaces.

In a recent manuscript by Estévez-Herrera et al. (2016), a relatively newly appreciated biophysical role of ATP is elucidated. Namely, the authors provide evidence using *in vitro* chromaffin cells that ATP agglomerates peptides and amines within the vesicles, thus reducing their particle numbers and effectively reducing the osmotic pressure within the vesicles. This may be critically important for maintaining isotonicity and survival of the vesicles in the cytosolic matrix.

ATP is a highly negatively charged molecule. It cannot simply diffuse through the membrane. Though the existence of a vesicular nucleotide transporter (VNUT) was predicted for a long time (Stiernet et al., 2006), the solute carrier protein SLC17A9 was only recently identified as the channel that transports ATP through the membrane (Sawada et al., 2008). Since its discovery, several studies have shown existence of SLC17A9 in enteric synaptic vesicles (Chaudhury et al., 2012), biliary epithelial microvesicles (Sathe et al., 2011), in the central nervous system (Larsson et al., 2012) and in the beta cells of the pancreas (Sakamoto et al., 2014).

In the beta cells of the pancreas, the insulin granules are not freely soluble molecules in the cytosol but rather exists as packaged vesicles. Electron micrographs of beta cells and physiological experiments have revealed that these insulin granules undergo regulated exocytosis to a glucose load, rather than random secretion (Dean, 1973). Potassium-sensitive ATP channels have been shown to play an important role for sensing of the glucose load and initiation of the exocytosis (Rutter and Hill, 2006).

Recently, it has been suggested that the exocytosis involving insulin granules resembles remarkable similarity to the pattern of inhibitory enteric neuromuscular neurotransmission (Chaudhury, 2014). Insulin granule exocytosis involves sequential release of ATP and the gaseous nitric oxide (NO), which may have important regulatory roles.

The SLC17A9 channel transports ATP within the insulin containing dense core vesicles (Sawada et al., 2008). In VNUT^−/−^ mice, the number and appearances of secretory granules in islet β-cells of knockout mice were normal (Sakamoto et al., 2014). However, insulin exocytosis was accelerated (Sakamoto et al., 2014) The recent manuscript by Estévez-Herrera et al. (2016) may potentially explain this significant observation. It is well known that insulin hypersecretion is an early stage pathophysiology which occurs in genetic obesity and prediabetes (Gonzalez et al., 2013; Irles et al., 2015). When VNUT is absent or deficient, ATP fails to enter the beta cells, or may load the vesicles suboptimally (Sakamoto et al., 2014; Figure 1). The decrease of ATP within the vesicle may alter the consistency of the dense crystalline core of the insulin. Thus, the vesicular osmotic pressure may be considerably elevated, leading to rapid exocytosis. Additionally, due to the lack of insulin polymer formation with ATP, the granular exocytosis may be sped up. These aspects remains to be examined.

**Figure 1:**
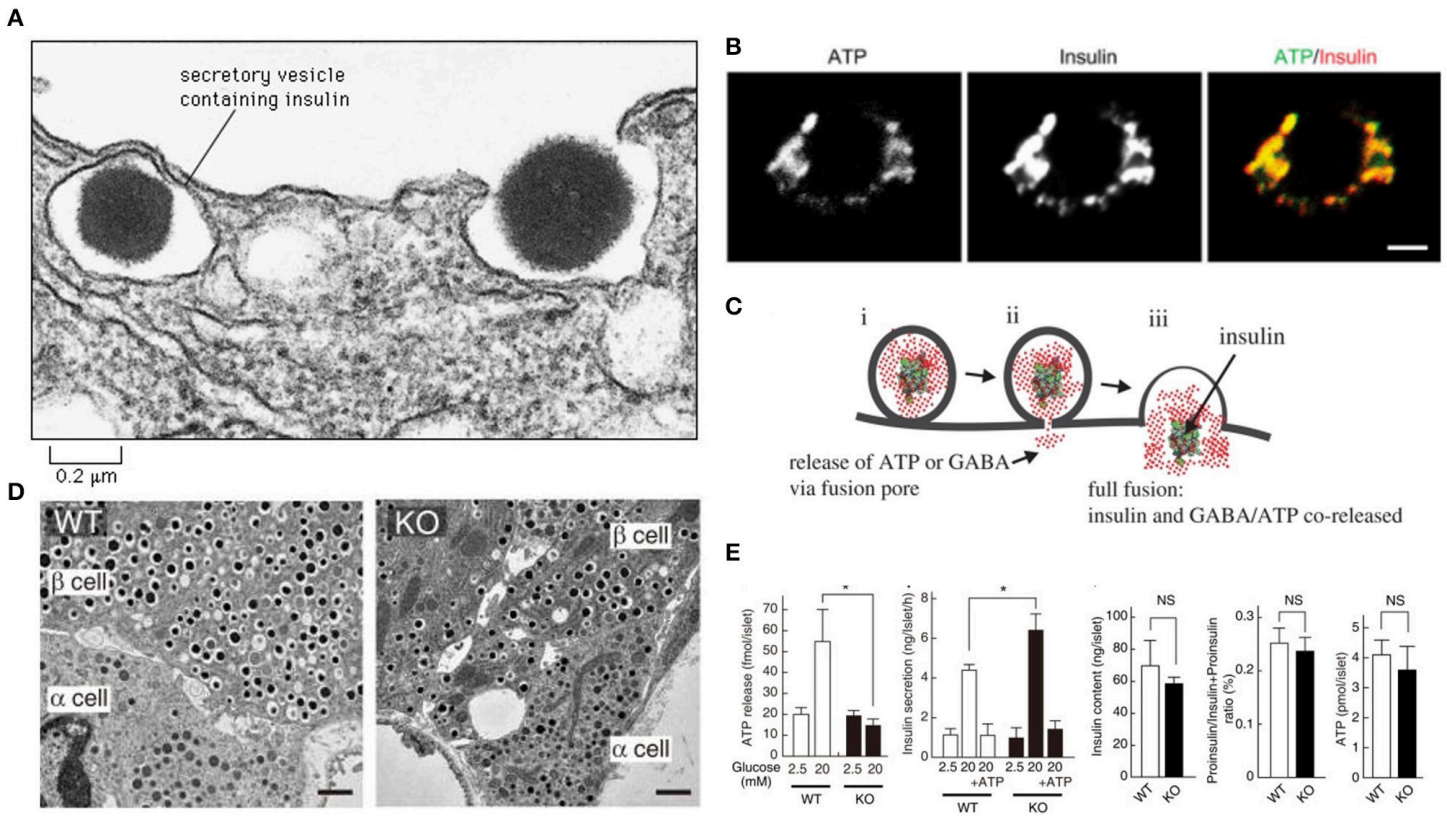
**ATP may contribute to colligative property of insulin containing vesicles (A)**. Note the exocytotic insulin vesicle and the dense insulin cluster in the electron micrograph. Recent study by Estévez-Herrera et al. (2016) suggest that ATP may contribute to colligative and osmotic balance of peptide containing vesicles. Such may be true for insulin vesicles **(B)**. ATP and insulin colocalize in beta cells of pancreas. Scale bar, 2 μm **(C)**. During exocytosis, both ATP and insulin are co-released. This co-release may have wide implications as discussed in the commentary **(D)**. In SLC17A9 knockout, the insulin containing vesicles are not altered in number or appearance **(E)**. In SLC17A9 knockout, the ATP and insulin contents of vesicles are decreased. But note that insulin exocytosis in SLC17A9 knockout is accelerated. This may occur due to lack of formation of ATP-(insulin)_n_ polymer. This rapid exocytosis is a feature of early prediabetic state, as well as seen in progressive type II diabetes mellitus. Reproduced with permission from Sakamoto et al. (2014) and Liu et al. (2014). ^*^Significance.

Type II diabetes mellitus is characterized by progressive exhaustion of the beta cells of the pancreas (Butler et al., 2016). This leads to increased insulin resistance and finally leads to an insulin-dependent state. The rates of beta cell damage are stochastic, and are likely multifactorial, including the effect of ongoing treatment with anti-diabetic medications. The ATP content in SLC17A9 knockout mice was reduced, though not significantly (Sakamoto et al., 2014; Figure 1). It may be remembered that insulin granule exocytosis is very complex and may involve several protein interactors, association with the cellular cytoskeleton, motor proteins like myosin Va, complex spatial negotiations at the subcellular cortex and finally the interaction with the membrane, where the dynamic pore size may be a critical determinant of exocytosis of the insulin, which is released either as a monomer or dimer (Ashcroft et al., 1973; Rothman, 1994; Li et al., 1995; Sudhof, 1995; Ivarsson et al., 2005; Braun et al., 2009; Karanauskaite et al., 2009; Wang and Thurmond, 2009; Solimena and Speier, 2010; Galvanovskis et al., 2011; MacDonald, 2011; Andersson et al., 2012; Schvartz et al., 2012; Seino, 2012; Rosengren et al., 2012; Wiseman and Thurmond, 2012; Kalwat and Thurmond, 2013; Moghadam and Jackson, 2013; Rorsman and Braun, 2013; Stamper and Wang, 2013; Xie et al., 2013; Bergeron et al., 2014; Gaisano, 2014; Heaslip et al., 2014; Satoh, 2014; Sun et al., 2014; Hoang Do and Thorn, 2015; Rutter et al., 2015; Schumacher et al., 2015; Takahashi, 2015; Takahashi et al., 2015; Wuttke, 2015; Thorn et al., 2016). Some or all of these pathways may be affected or altered in SLC17A9 channelopathy.

In this commentary, we advance the hypothesis that SLC17A9 dysfunction may contribute to the ongoing inefficacy of insulin exocytosis, leading to progressive diabetes. The lack of entry of ATP through a dysfunctional SLC17A9 (Sakamoto et al., 2014; Estévez-Herrera et al., 2016) may lead to a labile insulin vesicle formation, with enhanced rates of exocytosis. This may lead to increased demand on the cell for further insulin biogenesis, which may lead to a cascade of genomic stress and ultimate beta cell death. The mechanisms involved in the rapid dissolution of the insulin crystal before and during exocytosis, is not well-studied. Newer methodologies may be employed to study the insulin porosome (Jena, 2009).

What causes SLC17A9 dysfunction is not clear. Interestingly, SLC17A9 is gated by ATP itself (Sawada et al., 2008). Thus, ATP generation may be a limiting factor. Recent evidence suggest the inefficiency of mitochondrial coupling function with progressive diabetes, leading to diminished ATP production (Maechler, 2013; Peiris et al., 2016). This may be a potential underlying mechanism for the SLC17A9 dysfunction. Also, the channel may be gated by NO. NO downstream effectors are present in the beta cells and NO is known to inhibit ATP loading of dense vesicles (Machado et al., 2000). Using a range of biophysical methods including dynamic light scattering, turbidimetry, circular dichroism and atomic force microscopy, an elegant study has demonstrated that the amino acid L-arginine (Arg) has the ability to influence insulin aggregation propensity (Smirnova et al., 2015). The modification of the net charge of insulin induced by increase in the pH level of the incubation medium resulted in major alterations clustering of Arg-insulin. Using pH markers like pHluorin, vesicle pH may be assessed, which may serve as a surrogate for the vesicle osmotic pressure, which is otherwise a challenging technique to perform. Here, we may recollect that acidification of the vesicles is the main driver of ATP entry within the vesicles (Sawada et al., 2008).

There have been recent advances of novel markers that can tag synaptic proteins, especially those acting on the membranes. PET imaging may detect these global aspects of neurotransmission (Finnema et al., 2016). It is possible that such novel imaging may be applied for estimating beta cell mass in progressive diabetes mellitus. This is a huge area of unmet need in diabetes mellitus management. SLC17A9 may be tagged and imaged with similar novel methodologies to estimate whether their quantitative distribution is altered in progressive diabetes. Currently, C peptide analyses is used as a crude method to estimate disease status and functional beta cell reserve. Our thesis provides a novel paradigm in approaching management of diabetes mellitus from a precision medicine perspective.

## Author contributions

ArC, conceptualized and development of manuscript, drafted manuscript. CD, major intellectual contribution. VD, major intellectual contribution. CM, important discussion. NS, important discussion. WM, important discussion and conceptualizations. RR, important discussion. AM, important discussion, manuscript recheck. AdR, important discussion and prospects. All authors read and approved final version of manuscript.

### Conflict of interest statement

The authors declare that the research was conducted in the absence of any commercial or financial relationships that could be construed as a potential conflict of interest.
